# Ecological Risk Assessment of Metal Pollution along Greater Cairo Sector of the River Nile, Egypt, Using Nile Tilapia, *Oreochromis niloticus*, as Bioindicator

**DOI:** 10.1155/2015/167319

**Published:** 2015-11-04

**Authors:** Wael A. Omar, Wafai Z. A. Mikhail, Hanaa M. Abdo, Tarek A. Abou El Defan, Mamdouh M. Poraas

**Affiliations:** ^1^Department of Zoology, Faculty of Science, Cairo University, Giza 12613, Egypt; ^2^Department of Biology, Faculty of Science, Taif University, Taif 21944, Saudi Arabia; ^3^Department of Natural Resources, Institute of African Research and Studies, Cairo University, Giza 12613, Egypt; ^4^Soil, Water and Environment Research Institute (SWERI), Agricultural Research Center, Ministry of Agriculture and Land Reclamation, Giza 12613, Egypt

## Abstract

The present work aims to evaluate seasonal metal pollution along Greater Cairo sector of the River Nile, Egypt, using wild Nile tilapia, *Oreochromis niloticus*, as bioindicator and to conduct a risk assessment for human consumers. Greater Cairo is the largest populated area along the whole course of River Nile with a wide range of anthropogenic activities. Effects of metal pollution on fish body indices were studied using condition factor (CF) and scaled mass index (SMI). Metal pollution index (MPI) showed that the total metal load in fish organs followed the follwoing order: kidney > liver > gill > muscle which gives a better idea about the target organs for metal accumulation. Metal concentrations in fish muscle (edible tissue) showed the following arrangement: Fe > Zn > Cu > Mn > Pb > Cd. Metal's bioaccumulation factor (BAF) in fish muscle showed the following arrangement: Zn > Cu > Fe > Mn > Cd and Pb. The hazard index (HI) as an indicator of human health risks associated with fish consumption showed that adverse health effects are not expected to occur in most cases. However, the metals' cumulative risk effects gave an alarming sign specifically at high fish consumption rates.

## 1. Introduction

Greater Cairo is the largest metropolitan area in Egypt and Africa, the third largest urban area in the Islamic World after Jakarta and Karachi, and the world's 16th largest metropolitan area with a total population of about 18 million according to the 2006 census [1]. The uprising increase in modern industries and agricultural, touristic, and urbanization activities in this area may be considered as the main sources of pollution to both the aquatic environment and its coexisting ecosystems.

The aquatic environment makes up a major part of the environment and resources of the interested area. Therefore, its safety is directly related to human health. Pollution, loss of biodiversity, and habitat destruction are probably the main environmental threats for aquatic ecosystems. Moreover, the excessive contamination of aquatic ecosystems has evoked major environmental and health concerns worldwide [2, 3]. Pollutants can induce various biological responses in fish, affecting the organisms from the biochemical to the population-community levels [4, 5], and cause various harmful effects on wildlife [6]. Among the various toxic pollutants, trace metals represent a harmful group of elements due to their strong impact on stability of aquatic ecosystems, toxicity persistence, and accumulation tendency [7]. Among trace metals, some are potentially toxic (As, Cd, Pb, and Hg), others are probably essential (Ni, V, and Co), and many are essential (Cu, Zn, Fe, and Mn) [8]. Even essential metals can produce toxic effects when the metal intake is excessively elevated [9].

Fish are widely used in quality assessment of aquatic environment and as bioindicators of environmental pollution [10, 11]. So, field studies on early adverse effects of contaminants, measured directly in organisms in their natural environment, represent one of the main target areas in environmental biomonitoring programs [12]. Inland fisheries including River Nile yield the major part of Egyptian fish production where Egypt is ranked as 7th of the top ten countries in inland fish production [13]. The Nile tilapia,* Oreochromis niloticus,* is one of the most important economic fish species in Egypt and represents the species of consumers' choice with unquestionable market demand [14].

The present study aims to provide comparable data on seasonality of metal abundance in water and tissues of wild* O. niloticus* collected from four sites covering the whole Greater Cairo sector of the River Nile and to evaluate possible ecotoxicological human health risks associated with fish consumption. The selected sites are considered ideal to evaluate effects of anthropogenic activities concerning metal pollution up- and downstream River Nile.

## 2. Materials and Methods

### 2.1. Study Area

**Figure 1:**
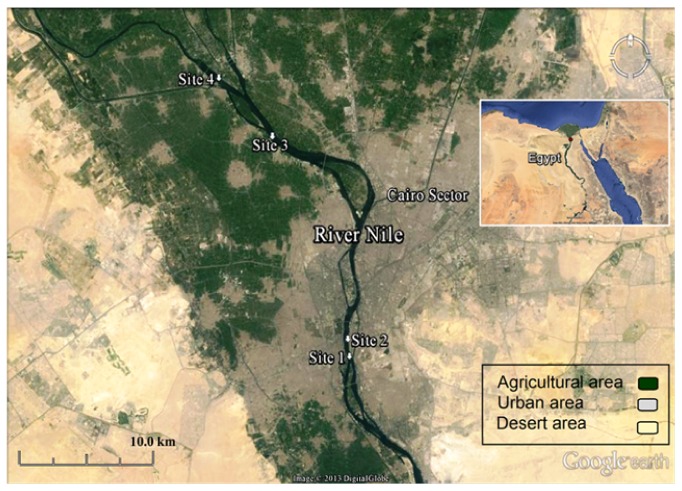
Map of Cairo sector showing the study sites (source: Google earth.com).

Surface water and wild male Nile tilapia (*O. niloticus*) samples were collected seasonally during 2013-2014 with the help of local fishermen from the following sites (Figure 1).

*Site 1 (Giza).* It is located upstream at inhabited island locally called Bein El-Bahreen island. Most of the population there are local fishermen and farmers with agricultural and domestic activities where untreated wastes are discharged directly to the River Nile. It is located at global positioning system (GPS) coordinates of 29° 59′ 9.20′′ N and 31° 13′ 16.19′′ E. 

*Site 2 (Manyal)*. It is located upstream about 2 km north of site 1 with normal domestic and touristic activities. It is located at GPS coordinates of 30° 0′ 54.56′′ N and 31° 13′ 17.62′′ E. 

*Site 3 (Imbaba)*. It is located downstream about 18 km north of site 1 and mostly dominated with industrial activities near the electric power station of West Cairo. It is located at GPS coordinates of 30° 8′ 41.13′′ N and 31° 9′ 41.98′′ E. 

*Site 4 (Kanater)*. It is located downstream about 24 km north of site 1. It is dominated with agricultural activities and lies in close proximity to El-Rahawy drainage canal which drains directly to the River Nile. It is located at GPS coordinates of 30° 10′ 54.98′′ N and 31° 7′ 8.24′′ E.

The selected sites cover a distance of 24 km along the course of River Nile. Water and fish samples were collected in a range of 500 m^2^ around the reported GPS coordinates of each site.

### 2.2. Water Sampling

Eight water samples were taken with a water sampler from each site seasonally. Duplicates of water samples were taken from four localities in each of the study sites between 10:00 and 12:00 a.m. at a depth of 30 cm below the water surface and stored at 4°C in clean 1000 mL sampling glass bottles according to Boyd [15].

### 2.3. Fish Sampling

A total number of 64 adult male* O. niloticus* fish of market size (16 fish/site) were collected seasonally from the study sites. Fish were transported in an icebox (0–4°C) to the laboratory. The body weight and total body length of each fish were measured; then fish were dissected to obtain muscle, liver, kidney, and gill samples. Tissue samples were processed for metal analysis immediately on the same day of sampling. Sexual differentiation was conducted visually during dissection and female fish were eliminated as they exhibit greater individual and seasonal fluctuations in length-weight relationship than males.

### 2.4. Fish Body Condition Indices

The condition factor (CF) was calculated according to Schreck and Moyle [16] as

(1) $$\begin{array}{c} CF=\left[\;\frac{Weight\left(g\right)}{{Length}^{3}\left(cm\right)}\;\right]\times 100. \end{array}$$

The scaled mass index (SMI) was calculated according to Peig and Green [17] as

(2) $$\begin{array}{c} \text{Scaled mass index}\;\left(\;SMI\;\right)=W_{i}{\left[\;\frac{L_{0}}{L_{i}}\;\right]}^{b_{SMA}}, \end{array}$$

where *W*_*i*_ and *L*_*i*_ are the weight (g) and length (cm) of each specimen, respectively, *L*_0_ is a suitable length to which the values were standardized (the arithmetic mean of the data set analyzed was used as *L*_0_), and *b*_SMA_ is the scaling exponent, that is, the slope of a standardized major axis (SMA) regression (also known as reduced major axis or RMA) of the mass-length relationship.

### 2.5. Metal Concentrations in Water and Fish Tissues

Metal concentrations (Cu, Zn, Mn, Cd, Pb, and Fe) were determined in water and fish tissues (muscle, liver, kidney, and gill) using flame atomic absorption spectrophotometer (Thermo Scientific ICE 3300, UK) provided with double beam and deuterium background corrector according to APHA [18]. All metal concentrations in fish tissues are reported in mg/kg dry weight, since dry weight rather than wet weight provides a more stable basis for comparison [19].

Tissue samples were dried at 105°C for 12 hours and then burned in a muffle furnace at 550°C for 16 hours. Samples were then acid digested and diluted with deionized water to known volume using the dry-ashing procedure proposed by Issac and Kerber [20] and Hseu [21].

#### 2.5.1. Quality Assurance and Quality Control (QA/QC) Procedures

The QA/QC protocols included the use of analytical blanks, replicate analyses, standard solutions prepared in the same acid matrix, and standard reference material. Standards for instrument calibration were prepared on the basis of monoelement certified reference solution (Merck). Standard reference material (Lake Superior fish 1946; National Institute of Standards and Technology (NIST), USA) was used to validate analysis, and the metal average recovery percentages ranged from 93% to 107% for all measured samples.

### 2.6. Statistical Analyses

The results were expressed as mean ± SE. Data were subjected to tests for normality and homogeneity. Test for normality was positive and followed the normal distribution; also test for homogeneity showed homogenous distribution of all data within the bell shape range. Data were statistically analyzed using analysis of variance (*F*-test) combined with Tukey's post hoc test to determine significant differences which are indicated by different case letters in the descending order A, B, C, and D at *P* < 0.05 using Statistical Analysis System, SAS, Version 9.1 [22].

### 2.7. Metal Pollution Index (MPI)

MPI was calculated to indicate the overall metal load in various fish tissues using the following formula according to Usero et al. [23]:

(3) $$\begin{array}{c} MPI={\left(\;M_{1}\times M_{2}\times M_{3}\times \cdots \times M_{n}\;\right)}^{1/n}, \end{array}$$

where *M*_*n*_ is the mean concentration of metal *n* (mg/kg dry wt.) in the examined tissue.

### 2.8. Bioaccumulation Factor (BAF)

BAF of trace metals in fish muscle was calculated according to Gobas et al. [24] as

(4) $$\begin{array}{c} BAF\left(\;L/kg\;\right)=\frac{C_{m}}{C_{w}}, \end{array}$$

where *C*_*m*_ is the mean metal concentration in fish muscle (mg/kg dry wt.) and *C*_*w*_ is the mean metal concentration in water (mg/L).

### 2.9. Human Risk Assessment

The following risk assessment procedures were conducted according to the United States Environmental Protection Agency (USEPA) [25]. The level of exposure resulting from oral consumption of trace metals in fish edible tissues was expressed by calculating the average daily dose (ADD; average daily intake of a specific chemical over a lifetime) using the following equation [25]:

(5) $$\begin{array}{c} ADD\left(\;mg/kg/day\;\right)=\frac{C_{m}\times IR\times EF\times ED}{BW\times AT}, \end{array}$$

where *C*_*m*_ is the mean metal concentration in fish muscle (mg/kg dry wt.), IR is the ingestion rate (0.0312 and 0.1424 kg/day for normal and habitual fish consumers, resp.), EF is the exposure frequency (365 days/year), ED is the exposure duration over a lifetime (assumed as 70 years), BW is the body weight (assumed as 70 kg for normal adults), and AT is the average lifetime (70 years × 365 days/year). Since ADD was calculated for a 70-year-old human, the reported equation was abbreviated to be

(6) $$\begin{array}{c} ADD\left(\;mg/kg/day\;\right)=\frac{C_{m}\times IR}{BW}. \end{array}$$

Risk was assessed by calculating the hazard index (HI; index of adverse health effects from intake of specific contaminant in food). HI is expressed as the ratio of the ADD to the oral reference dose of the metal according to the following equation proposed by USEPA [25]:

(7) $$\begin{array}{c} \text{Hazard Index}=\frac{ADD}{Oral\;\;RfD}, \end{array}$$

where oral RfD is the oral reference dose of the metal (mg/kg/day) based on the safe upper level of metal's oral intake for an adult human with average body weight of 70 kg. The oral RfD for Cu, Zn, Mn, Cd, and Fe is 0.04, 0.3, 0.14, 0.001, and 0.7 mg/kg/day, respectively [26], while that for Pb is 0.003 mg/kg/day [27]. HI values < 1.0 indicate that adverse health effects are not likely to occur. However, if the ADD of certain metal exceeds its oral RfD and thus the HI ≥ 1.0, it may be presumed that adverse health effects are expected to occur. The cumulative risk effect of all metals was calculated as the sum of HI values [28, 29].

## 3. Results and Discussion

Assessing morphological parameters is one of the most straightforward methods to study the effects of water contamination on fish because of the ease of recognition and examination when compared with other types of biomarkers [30]. Increasing the sample size by calculating the annual mean for each site and the seasonal mean among the study sites proved to be beneficial and gave a better idea about the measured parameters. The annual mean of both body total length and body weight showed the following arrangement: site 3 > site 1 > site 4 > site 2. Meanwhile, the seasonal mean of both parameters showed the following arrangement: summer > winter > spring > autumn (Table 1). Body condition is a sensitive and reliable endpoint in chronic toxicological investigations. In addition, certain body indices, such as the condition factor (CF), can provide information on potential pollution influence [31] but do not give information of specific responses to toxic substances in the media [32]. Condition factor is used to evaluate the well-being or fitness of fish as it is based on the hypothesis that heavier fish of a given length are of best condition. The pattern of seasonal variation in values of condition factor of* O. niloticus* coincides with that reported by Hirpo [33] for the same species in Lake Babogaya, Ethiopia, where he attributed this variation to seasonal fluctuations in environmental factors, food supply and quality, feeding rate, stressors, and reproductive activity. Toxic substances in the water may affect fish body condition by directly changing metabolism and increasing the energy required to maintain homeostasis, or they can indirectly impact growth by reducing food availability [34]. While body indices are not very sensitive and may be affected by other nonpollutant factors, they still serve as initial screening biomarkers to indicate exposure and its effects on fish [35].

Any fluctuation in CF values may reflect the health condition of fish as well as their body protein and lipid contents [36]. The scaled mass index (SMI) provides a novel indicator of ecosystem health and proved to be a better indicator of the relative size of energy reserves and other body components than the traditional CF [17, 37]. This is confirmed by the present findings where the pollution condition in the studied sites did not show significant effect on fish CF but the effect was clear on the SMI which showed significant decrease in sites 2 and 4. Generally, site 2 showed the lowest annual mean of both CF and SMI indicating the intricate pollution condition as well as the major effect of specific anthropogenic activities prevailing in this site (Table 1). Natural and anthropogenic disturbances are key forces governing the structure and functioning of aquatic communities. Understanding how these factors shape the organism performance can help to identify the most vulnerable species and develop effective management strategies [37]. The present findings indicate the minor effect of site variability on fish body condition indices and confirm that seasonality, including other related abiotic and biotic factors, affects greatly these indices.

A preliminary survey (unpublished data) was conducted in the study area to investigate levels of common metals. This survey indicated that the most common metals in the studied sites were copper, zinc, manganese, cadmium, lead, and iron. The metal pollution index (MPI) was used to indicate the total metal load in vital organs of* O. niloticus*. It is used to simplify the data and to provide it as one value instead of many for each studied organ when the measured metals are beyond five in number [38]. Results of MPI are given in Table 2. The sequence of MPI in different organs followed the following order: kidney > liver > gill > muscle which gives a better idea about the target organs for metal accumulation in* O. niloticus* fish especially when using the annual and seasonal means for that purpose. The results clearly indicate that each tissue has different capacity of metal accumulation. Generally, the highest values of MPI were recorded during spring season for all studied vital organs. Meanwhile, the lowest values of MPI were recorded during winter season for both fish muscle and liver samples but in case of kidney and gill samples they were recorded during summer season. Metals are not evenly distributed in the fish body but accumulate mainly in metabolically active tissues such as kidney, liver, and gill [39] while muscle shows the least metal accumulation that is mostly due to its low levels of binding proteins and enzymatic activities [40]. Fish accumulate metals both by ingestion of contaminated food and by contact of their respiratory surfaces with contaminated water [41]. Metal distribution among different tissues of aquatic organisms depends on the mode of exposure and can serve as a pollution indicator [42].

Table 3 reveals the metal concentrations in muscle of* O. niloticus* (mg/kg dry wt.) which are generally arranged in the following order: Fe > Zn > Cu > Mn > Pb > Cd. This arrangement reflects the essentiality of these metals for fish body functions. Some of these metals are classified biochemically as essential elements in the bodies of living organisms and aquatic plants (such as Fe, Zn, and Cu) when present in trace amounts but when they are present in high concentrations they become toxic [43]. The calculated bioaccumulation factor for different metals in fish muscle gives a clear image about the concentration of these metals in fish muscle relative to their concentration in water and the affinity of fish muscle to accumulate these metals relative to their abundance in water.

As indicated in Table 4, metal concentrations in fish muscle were several fold higher than their concentrations in water. The BAF in fish muscle shows the following arrangement: Zn > Cu > Fe > Mn > Cd and Pb. There was a great fluctuation in BAF of both Cd and Pb which were the least accumulated metals in fish muscle. Generally, BAF of Cd was higher than Pb during summer season along all sites and the case was reversed during autumn season whereas during winter and spring seasons its abundance was site dependent. This clearly indicates the effect of seasonality of natural or anthropogenic sources on metal abundance in aquatic media as well as the great effect of point and nonpoint sources of pollution along the study sites. Because many fish stay in rather confined regions of the river, they will suffer by one way or another if this aquatic system is contaminated by toxic substances [44].

Bioaccumulation of trace metals in tissues of aquatic organisms has been identified as an indirect measure of the abundance and availability of these metals in the environment [45]. For this reason, monitoring fish tissue contamination represents an important function as an early warning of related water contamination problems and enables us to take appropriate action to protect public health and the environment [46].

Because fish respond with great sensitivity to changes in the aquatic environment, they are one of the most indicative factors in aquatic environment for the estimation of metal pollution and risk potential of human consumption [47]. Hence, it is important to determine metal concentrations in edible tissues of commercial fish in order to evaluate the possible risk of fish consumption [48].

The calculated hazard index (HI) is an integrated risk calculation package that combines both the metal level in fish edible tissues and the human consumption rate of these tissues to perform a risk characterization. In hazard identification, available data on biological endpoints are used to determine if a material is likely to pose a hazard to human health. These data are also used to define the type of potential hazard. In the dose-response assessment, data are used to estimate the amount of material that may produce a given effect in humans. The risk assessor may calculate a quantitative dose-response relationship and make it applicable for low-dose exposure, often by applying mathematical models to the data [49].

Fish consumption information is essential for assessing the human health implications associated with the consumption of chemically contaminated fish [50]. The calculated HI for detected metals, as indicated in Table 5, did not pose unacceptable risks at both proposed ingestion rates (for normal and habitual fish consumers) except for Pb in sites 1 and 2 for habitual fish consumers during spring season. Both ingestion rates used in the present study were reported by USEPA [25] for generalized human population but if the normal ingestion rate of 0.0435 kg/day proposed by FAO [51] specifically for adult Egyptians was used in the present HI calculations, the resulting HI values at normal ingestion rate would be even higher by a factor of 1.4 than the present findings. This clearly indicates the significance of incorporating the consumption rates in contaminants' risk assessments. The metals' cumulative risk effects showed unacceptable risks for habitual fish consumers during spring and winter seasons in site 2 as well as spring and summer seasons in site 1. Generally, sites 1 and 2 were the most polluted sites especially during spring season which shows that irregular domestic and touristic activities that prevail in these upstream sites during favorable weather conditions had major effects on water pollution rather than the regular agricultural and industrial activities that prevail in the other downstream sites all over the year.

## 4. Conclusion

The effect of different anthropogenic activities on metal load of fish edible tissues along the study period in all sites was evident and it was proved using the calculated MPI and BAF and specially the HI. Despite the low human health hazards expected due to consumption of each metal separately, the fish edible tissues contain totally abundant quantity of different metals which may lead to human adverse health effects. That is to say, the metals' cumulative risk effects gave an alarming sign. The present study affirms that application of the proposed human risk assessment (dose and consumption dependent variables) is more reliable in predicting the hazards posed on human consumers rather than the use of regular known permissible levels or the upper level of intake in food for human consumption which are not consumption dependent variables. Regular metal assessment surveys and advisories for fish consumption are recommended for water bodies around densely populated areas like Greater Cairo.

## Figures and Tables

**Table 1:** Body total length, body weight, condition factor, and scaled mass index of *O. niloticus*.

	Body total length (cm)					Body weight (g)				
	Site 1 (G)	Site 2 (M)	Site 3 (I)	Site 4 (K)	Seasonal mean	Site 1 (G)	Site 2 (M)	Site 3 (I)	Site 4 (K)	Seasonal mean
Winter	17.00 ± 0.71^aB^	13.33 ± 0.88^bB^	18.30 ± 0.93^aB^	14.23 ± 0.07^bB^	15.72	116.54 ± 11.06^aA^	49.88 ± 13.20^bB^	132.88 ± 14.84^aB^	63.57 ± 7.97^bB^	90.72
Spring	13.60 ± 0.32^cC^	13.53 ± 0.22^cB^	18.80 ± 0.36^aAB^	15.40 ± 0.40^bA^	15.33	45.22 ± 4.05^cB^	44.84 ± 0.56^cB^	161.40 ± 7.51^aAB^	75.56 ± 2.02^bA^	81.76
Summer	20.33 ± 0.88^abA^	16.30 ± 0.47^bcA^	23.20 ± 2.58^aA^	15.57 ± 0.23^cA^	18.85	153.90 ± 22.83^aA^	94.52 ± 6.08^bA^	192.76 ± 22.06^aA^	76.44 ± 4.21^bA^	129.41
Autumn	13.30 ± 0.21^aC^	14.60 ± 1.05^aAB^	14.90 ± 0.31^aB^	14.33 ± 0.17^aB^	14.28	50.68 ± 2.85^aB^	67.83 ± 17.25^aAB^	71.98 ± 4.31^aC^	59.53 ± 2.12^aB^	62.51
Annual mean	16.06	14.44	18.80	14.88	16.05^#^	91.59	64.27	139.76	68.78	91.10^#^

	Condition factor (CF) (%)					Scaled mass index (SMI) (g)				
	Site 1 (G)	Site 2 (M)	Site 3 (I)	Site 4 (K)	Seasonal mean	Site 1 (G)	Site 2 (M)	Site 3 (I)	Site 4 (K)	Seasonal mean

Winter	2.37 ± 0.15^aA^	2.01 ± 0.14^aAB^	2.16 ± 0.08^aAB^	2.20 ± 0.05^aA^	2.19	116.26 ± 5.94^bA^	47.74 ± 2.53^dA^	131.90 ± 0.20^aAB^	63.54 ± 1.27^cA^	89.86
Spring	1.79 ± 0.06^cB^	1.81 ± 0.07^bcB^	2.43 ± 0.07^aA^	2.08 ± 0.12^bA^	2.01	76.22 ± 3.23^bC^	42.54 ± 2.18^cA^	151.73 ± 3.57^aA^	45.96 ± 3.41^cBC^	79.11
Summer	1.80 ± 0.06^aB^	2.18 ± 0.07^aA^	1.65 ± 0.31^aB^	2.02 ± 0.04^aA^	1.91	99.27 ± 5.21^aB^	44.52 ± 2.25^bA^	115.96 ± 4.29^aB^	42.25 ± 2.22^bC^	75.50
Autumn	2.15 ± 0.05^aA^	2.09 ± 0.08^aAB^	2.18 ± 0.06^aAB^	2.02 ± 0.02^aA^	2.11	90.38 ± 2.49^bBC^	46.43 ± 2.09^dA^	114.62 ± 3.10^aB^	56.91 ± 2.49^cAB^	77.09
Annual mean	2.01	2.02	2.11	2.08	2.05^#^	95.53	45.31	128.55	52.17	80.39^#^

Data are represented as means of 16 samples ± SE.

Statistically significant differences (*P* < 0.05) are shown with different superscript lowercase letters in the same raw and different superscript capital letters in the same column for each of the measured parameters.

Mean body total length value was used as *L*_0_ for calculating the scaled mass index.

G, Giza; M, Manyal; I, Imbaba; and K, Kanater.

^#^The mean of all measurements for each parameter.

**Table 2:** Metal pollution index (MPI) of total trace metals in vital organs of *O. niloticus*.

	MPI				
	Site 1 (G)	Site 2 (M)	Site 3 (I)	Site 4 (K)	Seasonal mean
Muscle					
Winter	1.34	2.45	1.29	1.59	1.67
Spring	5.85	3.14	2.59	2.47	3.51
Summer	1.87	2.11	1.38	2.17	1.88
Autumn	2.10	1.76	2.26	1.48	1.90
Annual mean	2.79	2.37	1.88	1.93	2.24^#^
Liver					
Winter	20.62	21.69	25.40	11.02	19.68
Spring	52.05	16.36	13.51	17.78	24.93
Summer	16.03	27.41	14.47	32.73	22.66
Autumn	23.75	19.38	25.00	18.82	21.74
Annual mean	28.11	21.21	19.59	20.09	22.25^#^
Kidney					
Winter	31.82	38.39	16.90	17.59	26.17
Spring	82.11	38.00	37.74	32.76	47.66
Summer	42.89	23.15	11.11	25.19	25.59
Autumn	22.54	24.01	23.66	35.95	26.54
Annual mean	44.84	30.89	22.35	27.87	31.49^#^
Gill					
Winter	10.15	11.70	4.95	7.15	8.48
Spring	10.83	13.74	8.37	8.77	10.43
Summer	5.96	7.63	5.49	7.49	6.64
Autumn	9.59	6.10	10.49	7.11	8.32
Annual mean	9.13	9.79	7.32	7.63	8.47^#^

G, Giza; M, Manyal; I, Imbaba; and K, Kanater.

^#^The mean of all measurements for each organ.

**Table 3:** Metal concentrations in muscle of* O. niloticus* (mg/kg dry wt.).

	Cu					Zn				
	Site 1 (G)	Site 2 (M)	Site 3 (I)	Site 4 (K)	Seasonal mean	Site 1 (G)	Site 2 (M)	Site 3 (I)	Site 4 (K)	Seasonal mean
Winter	5.53 ± 1.23^aA^	4.02 ± 0.13^abAB^	2.66 ± 0.32^bA^	3.24 ± 0.34^bA^	3.86	25.57 ± 0.86^aB^	29.45 ± 1.24^aB^	25.84 ± 2.32^aAB^	27.83 ± 3.55^aA^	27.17
Spring	8.89 ± 0.72^aB^	7.82 ± 2.32^abA^	4.36 ± 0.65^bA^	4.67 ± 0.29^abA^	6.44	55.81 ± 6.49^aA^	42.68 ± 3.69^abA^	32.82 ± 5.68^bA^	30.93 ± 2.54^bA^	40.56
Summer	4.37 ± 0.73^aB^	2.19 ± 0.43^aB^	2.67 ± 0.28^aA^	3.15 ± 1.82^aA^	3.10	33.74 ± 1.39^aB^	32.16 ± 0.27^aB^	20.48 ± 0.68^cB^	27.71 ± 2.21^bA^	28.52
Autumn	5.90 ± 0.62^aB^	4.70 ± 0.10^aAB^	3.00 ± 0.71^bA^	2.37 ± 0.17^bA^	3.99	27.83 ± 1.86^abB^	25.55 ± 1.73^abB^	22.61 ± 1.73^bAB^	28.56 ± 1.45^aA^	26.14
Annual mean	6.17	4.68	3.17	3.36	4.35^#^	35.74	32.46	25.44	28.76	30.60^#^

	Mn					Cd				
	Site 1 (G)	Site 2 (M)	Site 3 (I)	Site 4 (K)	Seasonal mean	Site 1 (G)	Site 2 (M)	Site 3 (I)	Site 4 (K)	Seasonal mean

Winter	2.92 ± 0.68^aAB^	1.62 ± 0.69^aA^	2.74 ± 0.72^aA^	1.60 ± 0.31^aAB^	2.22	0.01 ± 0.01^aB^	0.08 ± 0.04^aA^	0.03 ± 0.01^aB^	0.01 ± 0.01^aB^	0.033
Spring	4.45 ± 0.34^aA^	0.81 ± 0.26^cA^	2.49 ± 0.74^bA^	2.46 ± 0.45^bA^	2.55	0.08 ± 0.04^aA^	0.05 ± 0.04^aA^	0.05 ± 0.01^aA^	0.07 ± 0.02^aA^	0.063
Summer	0.36 ± 0.03^bC^	1.06 ± 0.30^aA^	0.89 ± 0.08^aA^	1.09 ± 0.06^aB^	0.85	0.05 ± 0.01^aAB^	0.07 ± 0.02^aA^	0.06 ± 0.01^aA^	0.09 ± 0.01^aA^	0.068
Autumn	1.49 ± 0.69^aBC^	1.12 ± 0.18^aA^	3.10 ± 2.08^aA^	1.79 ± 0.23^aAB^	1.88	0.05 ± 0.01^aAB^	0.02 ± 0.01^bA^	0.05 ± 0.01^aA^	0.02 ± 0.01^bB^	0.034
Annual mean	2.31	1.15	2.31	1.74	1.87^#^	0.048	0.053	0.048	0.048	0.049^#^

	Pb					Fe				
	Site 1 (G)	Site 2 (M)	Site 3 (I)	Site 4 (K)	Seasonal mean	Site 1 (G)	Site 2 (M)	Site 3 (I)	Site 4 (K)	Seasonal mean

Winter	0.05 ± 0.03^aB^	0.53 ± 0.37^aB^	0.03 ± 0.01^aC^	0.36 ± 0.05^aAB^	0.24	28.46 ± 7.72^aB^	26.90 ± 7.25^aA^	27.44 ± 9.31^aA^	31.04 ± 5.55^aB^	28.46
Spring	1.56 ± 0.52^aA^	2.07 ± 0.32^aA^	0.24 ± 0.04^bB^	0.18 ± 0.04^bB^	1.01	145.03 ± 4.21^aA^	34.26 ± 5.24^bA^	71.36 ± 33.82^bA^	50.19 ± 8.04^bA^	75.21
Summer	0.90 ± 0.25^aAB^	0.53 ± 0.06^abB^	0.17 ± 0.05^abB^	0.48 ± 0.05^bA^	0.52	17.70 ± 0.84^bB^	32.02 ± 2.81^aA^	13.76 ± 1.70^bA^	25.69 ± 2.36^aB^	22.29
Autumn	0.24 ± 0.08^abB^	0.43 ± 0.07^aB^	0.46 ± 0.02^aA^	0.15 ± 0.10^bB^	0.32	29.20 ± 2.90^aB^	26.11 ± 2.20^aA^	27.61 ± 6.58^aA^	28.65 ± 11.95^aB^	27.89
Annual mean	0.69	0.89	0.23	0.29	0.52^#^	55.10	29.82	35.04	33.89	38.46^#^

Data are represented as means of 16 samples ± SE.

Statistically significant differences (*P* < 0.05) are shown with different superscript lowercase letters in the same raw and different superscript capital letters in the same column for each of the measured metals.

G, Giza; M, Manyal; I, Imbaba; and K, Kanater.

^#^The mean of all measurements for each metal.

**Table 4:** Bioaccumulation factor (BAF) of trace metals (l/kg) in muscle of* O. niloticus*.

	Cu					Zn				
	Site 1 (G)	Site 2 (M)	Site 3 (I)	Site 4 (K)	Seasonal mean	Site 1 (G)	Site 2 (M)	Site 3 (I)	Site 4 (K)	Seasonal mean
Winter	204.81	365.45	166.25	294.55	257.77	799.06	920.31	891.03	1070.38	920.20
Spring	987.78	372.38	189.57	137.35	421.77	2325.42	1940.00	1562.86	1472.86	1825.28
Summer	150.69	68.44	70.26	108.62	99.50	992.35	846.32	512.00	589.57	735.06
Autumn	393.33	470.00	214.29	197.50	318.78	1637.06	1502.94	1739.23	2596.36	1868.90
Annual mean	434.15	319.07	160.09	184.50	274.45^#^	1438.47	1302.39	1176.28	1432.29	1337.36^#^

	Mn					Cd				
	Site 1 (G)	Site 2 (M)	Site 3 (I)	Site 4 (K)	Seasonal mean	Site 1 (G)	Site 2 (M)	Site 3 (I)	Site 4 (K)	Seasonal mean

Winter	78.92	49.09	124.55	48.48	75.26	2.50	26.67	6.00	3.33	9.63
Spring	134.85	25.31	99.60	74.55	83.58	40.00	16.67	16.67	35.00	27.08
Summer	13.33	28.65	32.96	47.39	30.58	50.00	23.33	60.00	90.00	55.83
Autumn	59.60	41.48	155.00	66.30	80.59	12.50	6.67	12.50	6.67	9.58
Annual mean	71.68	36.13	103.03	59.18	67.50^#^	26.25	18.33	23.79	33.75	25.53^#^

	Pb					Fe				
	Site 1 (G)	Site 2 (M)	Site 3 (I)	Site 4 (K)	Seasonal mean	Site 1 (G)	Site 2 (M)	Site 3 (I)	Site 4 (K)	Seasonal mean

Winter	5.56	40.77	2.31	27.69	19.08	40.89	69.87	114.33	197.71	105.70
Spring	60.00	69.00	10.00	7.20	36.55	356.34	96.24	216.24	90.27	189.77
Summer	33.33	17.67	6.80	18.46	19.07	56.55	92.81	40.47	61.46	62.82
Autumn	18.46	30.71	25.56	13.64	22.09	161.33	123.16	233.98	236.78	188.81
Annual mean	29.34	39.54	11.17	16.75	24.20^#^	153.78	95.52	151.26	146.55	136.78^#^

G, Giza; M, Manyal; I, Imbaba; and K, Kanater.

^#^The mean of all measurements for each metal.

**Table 5:** Hazard index (HI) and the cumulative risk effect for normal and habitual fish consumers.

		Cu				Zn			
		Site 1 (G)	Site 2 (M)	Site 3 (I)	Site 4 (K)	Site 1 (G)	Site 2 (M)	Site 3 (I)	Site 4 (K)
Winter	Normal	0.06	0.04	0.03	0.04	0.04	0.04	0.04	0.04
	Habitual	0.28	0.20	0.14	0.16	0.17	0.20	0.18	0.19

Spring	Normal	0.10	0.09	0.05	0.05	0.08	0.06	0.05	0.05
	Habitual	0.45	0.40	0.22	0.24	0.38	0.29	0.22	0.21

Summer	Normal	0.05	0.02	0.03	0.04	0.05	0.05	0.03	0.04
	Habitual	0.22	0.11	0.14	0.16	0.23	0.22	0.14	0.19

Autumn	Normal	0.07	0.05	0.03	0.03	0.04	0.04	0.03	0.04
	Habitual	0.30	0.24	0.15	0.12	0.19	0.17	0.15	0.19

		Mn				Cd			
		Site 1 (G)	Site 2 (M)	Site 3 (I)	Site 4 (K)	Site 1 (G)	Site 2 (M)	Site 3 (I)	Site 4 (K)

Winter	Normal	0.01	0.01	0.01	0.01	0.004	0.04	0.01	0.004
	Habitual	0.04	0.02	0.04	0.02	0.02	0.16	0.06	0.02

Spring	Normal	0.01	0.003	0.01	0.01	0.04	0.02	0.02	0.03
	Habitual	0.06	0.01	0.04	0.04	0.16	0.10	0.10	0.14

Summer	Normal	0.001	0.003	0.003	0.003	0.02	0.03	0.03	0.04
	Habitual	0.01	0.02	0.01	0.02	0.10	0.14	0.12	0.18

Autumn	Normal	0.005	0.004	0.01	0.01	0.02	0.01	0.02	0.01
	Habitual	0.02	0.02	0.05	0.03	0.10	0.04	0.10	0.04

		Pb				Fe			
		Site 1 (G)	Site 2 (M)	Site 3 (I)	Site 4 (K)	Site 1 (G)	Site 2 (M)	Site 3 (I)	Site 4 (K)

Winter	Normal	0.01	0.08	0.004	0.05	0.02	0.02	0.02	0.02
	Habitual	0.03	0.36	0.02	0.24	0.08	0.08	0.08	0.09

Spring	Normal	0.23	0.31	0.04	0.03	0.09	0.02	0.05	0.03
	Habitual	1.06^*∗*^	1.40^*∗*^	0.16	0.12	0.42	0.10	0.21	0.15

Summer	Normal	0.13	0.08	0.03	0.07	0.01	0.02	0.01	0.02
	Habitual	0.61	0.36	0.12	0.33	0.05	0.09	0.04	0.07

Autumn	Normal	0.04	0.06	0.07	0.02	0.02	0.02	0.02	0.02
	Habitual	0.16	0.29	0.31	0.10	0.08	0.08	0.08	0.08

		Cumulative risk effect							
		Site 1 (G)		Site 2 (M)		Site 3 (I)		Site 4 (K)	

Winter	Normal	0.14		0.23		0.11		0.16	
	Habitual	0.63		1.03^*∗*^		0.51		0.73	

Spring	Normal	0.56		0.50		0.21		0.20	
	Habitual	2.54^*∗*^		2.30^*∗*^		0.95		0.89	

Summer	Normal	0.27		0.21		0.12		0.21	
	Habitual	1.22^*∗*^		0.94		0.56		0.95	

Autumn	Normal	0.19		0.18		0.19		0.12	
	Habitual	0.86		0.84		0.84		0.57	

G, Giza; M, Manyal; I, Imbaba; and K, Kanater.

^*∗*^HI ≥ 1.0, which is the point at which adverse health effects are expected to occur.
